# 1-(5a,5b,8,8,11a,13b-Hexamethyl­eicosa­hydro-1*H*-cyclo­penta­[*a*]chrysen-3-yl)-1-ethanone

**DOI:** 10.1107/S1600536808006831

**Published:** 2008-03-20

**Authors:** Altaf Hussain, Hamid Latif Siddiqui, Rehana Rashid, Khalid Mehmood Khan, Masood Parvez

**Affiliations:** a Institute of Chemistry, University of the Punjab, Lahore, Pakistan; b Department of Chemistry, University of Balochistan, Quetta, Pakistan; c International Center for Chemical Sciences, HEJ Research Institute of Chemistry, University of Karachi, Karachi-75270, Pakistan; d Department of Chemistry, The University of Calgary, 2500 University Drive NW, Calgary, Alberta, Canada T2N 1N4

## Abstract

The title compound, C_29_H_48_O, is a triterpenoid isolated from *Adiantum incisum forssk*. In the crystal structure, the asymmetric unit contains two independent mol­ecules which are not significantly different. Each mol­ecule contains four six-membered rings, all adopting chair conformations, and a five-membered ring in an envelope conformation. In the mol­ecular structure, non-classical intra­molecular C—H⋯O hydrogen bonds are observed.

## Related literature

For related literature, see: Ageta & Iwata (1966 ▶); Brahmachari *et al.* (2003 ▶); Bresciani *et al.* (2003 ▶); Hussain *et al.* (2007 ▶, 2008 ▶); Nadkarni & Nadkarni (1982 ▶); Reddy *et al.* (2001 ▶).
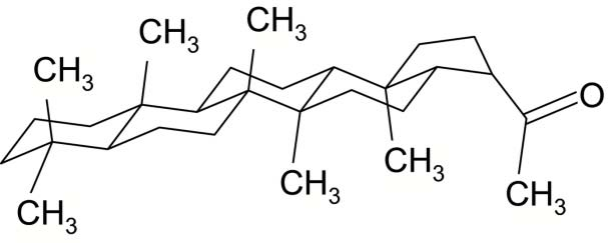


## Experimental

### 

#### Crystal data

C_29_H_48_O
*M* *_r_* = 412.67
Triclinic, 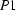

*a* = 6.581 (3) Å
*b* = 7.260 (4) Å
*c* = 27.908 (14) Å
α = 84.98 (2)°
β = 86.48 (3)°
γ = 64.01 (3)°
*V* = 1193.6 (10) Å^3^
*Z* = 2
Mo *K*α radiation
μ = 0.07 mm^−1^
*T* = 173 (2) K
0.12 × 0.10 × 0.05 mm

#### Data collection

Nonius KappaCCD diffractometer
Absorption correction: multi-scan (**SORTAV**; Blessing, 1997 ▶) *T*_min_ = 0.992, *T*_max_ = 0.997
7958 measured reflections
4272 independent reflections
3161 reflections with *I* > 2σ(*I*)
*R*_int_ = 0.050

#### Refinement

*R*[*F* ^2^ > 2σ(*F* ^2^)] = 0.069
*wR*(*F* ^2^) = 0.177
*S* = 1.05
4272 reflections
555 parameters
3 restraints
H-atom parameters constrained
Δρ_max_ = 0.25 e Å^−3^
Δρ_min_ = −0.30 e Å^−3^

### 

Data collection: *COLLECT* (Hooft, 1998 ▶); cell refinement: *DENZO* (Otwinowski & Minor, 1997 ▶); data reduction: *SCALEPACK* (Otwinowski & Minor, 1997 ▶); program(s) used to solve structure: *SAPI91* (Fan, 1991 ▶); program(s) used to refine structure: *SHELXL97* (Sheldrick, 2008 ▶); molecular graphics: *ORTEPII* (Johnson, 1976 ▶); software used to prepare material for publication: *SHELXL97*.

## Supplementary Material

Crystal structure: contains datablocks global, I. DOI: 10.1107/S1600536808006831/is2277sup1.cif

Structure factors: contains datablocks I. DOI: 10.1107/S1600536808006831/is2277Isup2.hkl

Additional supplementary materials:  crystallographic information; 3D view; checkCIF report

## Figures and Tables

**Table 1:** Hydrogen-bond geometry (Å, °)

*D*—H⋯*A*	*D*—H	H⋯*A*	*D*⋯*A*	*D*—H⋯*A*
C20—H20*B*⋯O30	0.99	2.37	2.832 (7)	108
C50—H50*B*⋯O60	0.99	2.39	2.837 (7)	107

**Fig. 1.**
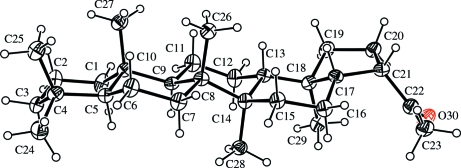
ORTEPII (Johnson, 1976) drawing of molecule A with displacement ellipsoids plotted at the 30% probability level.

**Fig. 2.**
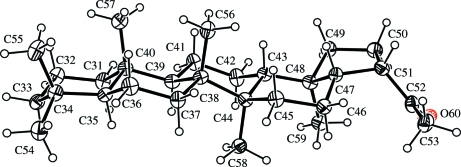
ORTEPII (Johnson, 1976) drawing of molecule B with displacement ellipsoids plotted at the 30% probability level.

## supplementary crystallographic information

### Comment

Triterpenoids are an important class of natural products and are recognized for their ethnobotanical nature *i.e.* analgesic, anti-microbial and anti-inflammatory (Brahmachari *et al.*, 2003; Bresciani *et al.*, 2003; Reddy *et al.*, 2001). *Adiantum incisum forssk*, *syn. Adiantum caudatum L.*, a fern, used in ayurvedic medicines, is well known for its use against diabetes and skin diseases (Nadkarni & Nadkarni, 1982). During its phytochemical investigations, the title compound (I), along with other compounds (Hussain *et al.*, 2007, 2008), was isolated and its crystal structure is hereby reported; it has been reported as one of the constituents of *Adiantum pedatum* (Ageta & Iwata, 1966).

The asymmetric unit of (I) contains two independent molecules A and B, presented in Figs. 1 and 2, respectively. There are no significant differences in the molecular dimensions of the two molecules. Both molecules contain four six-membered rings, all adopting chair conformations and a five-membered ring adopting envelope conformation with atoms C18 and C48, 0.656 (9) and 0.684 (9) Å out of the planes formed by the remaining ring atoms, C17/C19/C20/C21 and C47/C49/C50/C51, respectively. The acetyl groups bonded to the five membered rings show intramolecular H-bonding interactions, C20—H20···O30 and C50—H50···O60 (Table 1).

### Experimental

*Adiantum incisum forssk* was collected from Murree hills, Pakistan, during September 2003. After shade drying, pinnae were separated from rachis. The dried rhizomes, stipes and rachis (2.9 kg) were soaked in ethanol (20 *L*) for fifteen days and filtered. The filtrate was concentrated at 313 K under reduced pressure to give 51.33 g of ethanolic extract, which was subjected to silica-gel column chromatography eluting gradient solvent system hexane-chloroform-methanol. More than 300 fractions were monitored by thin layer chromatography and combined to give 38 main fractions. Fraction 5 was further purified by silica-gel column chromatography with hexane-chloroform (10:1) and preparative TLC with dichloromethane and petroleum ether (2:3) to give the pure title compound (I). X-ray quality crystals were grown from a solution of hexane-chloroform (9:1) at room temperature.

### Refinement

H atoms were included in the refinements at geometrically idealized positions with C—H distances 0.95 - 1.00 Å, and with *U*_iso_(H) = 1.2*U*_eq_(C), allowing for free rotation of the methyl groups. The final difference map was free of any chemically significant features. In the absence of significant anomalous scattering effects, an absolute structure could not be established in this analysis and therefore, Friedel pairs were merged.

### Crystal data

**Table:**

C_29_H_48_O	*Z* = 2
*M**_r_* = 412.67	*F*_000_ = 460
Triclinic, *P*1	*D*_x_ = 1.148 Mg m^−^^3^
Hall symbol: P 1	Melting point = 495–497 K
*a* = 6.581 (3) Å	Mo *K*α radiation λ = 0.71073 Å
*b* = 7.260 (4) Å	Cell parameters from 1724 reflections
*c* = 27.908 (14) Å	θ = 3.1–25.3º
α = 84.98 (2)º	µ = 0.07 mm^−^^1^
β = 86.48 (3)º	*T* = 173 (2) K
γ = 64.01 (3)º	Plate, colorless
*V* = 1193.6 (10) Å^3^	0.12 × 0.10 × 0.05 mm

### Data collection

**Table:**

Nonius KappaCCD diffractometer	4272 independent reflections
Radiation source: fine-focus sealed tube	3161 reflections with *I* > 2σ(*I*)
Monochromator: graphite	*R*_int_ = 0.050
*T* = 173(2) K	θ_max_ = 25.3º
ω and φ scans	θ_min_ = 3.1º
Absorption correction: multi-scan(*SORTAV*; Blessing, 1997)	*h* = −7→7
*T*_min_ = 0.992, *T*_max_ = 0.997	*k* = −8→8
7958 measured reflections	*l* = −33→33

### Refinement

**Table:**

Refinement on *F*^2^	Secondary atom site location: difference Fourier map
Least-squares matrix: full	Hydrogen site location: inferred from neighbouring sites
*R*[*F*^2^ > 2σ(*F*^2^)] = 0.069	H-atom parameters constrained
*wR*(*F*^2^) = 0.177	*w* = 1/[σ^2^(*F*_o_^2^) + (0.08*P*)^2^ + 0.9*P*] where *P* = (*F*_o_^2^ + 2*F*_c_^2^)/3
*S* = 1.05	(Δ/σ)_max_ = 0.003
4272 reflections	Δρ_max_ = 0.25 e Å^−^^3^
555 parameters	Δρ_min_ = −0.30 e Å^−^^3^
3 restraints	Extinction correction: none
Primary atom site location: structure-invariant direct methods	

### Special details

**Table:**

Geometry. All e.s.d.'s (except the e.s.d. in the dihedral angle between two l.s. planes) are estimated using the full covariance matrix. The cell e.s.d.'s are taken into account individually in the estimation of e.s.d.'s in distances, angles and torsion angles; correlations between e.s.d.'s in cell parameters are only used when they are defined by crystal symmetry. An approximate (isotropic) treatment of cell e.s.d.'s is used for estimating e.s.d.'s involving l.s. planes.
Refinement. Refinement of *F*^2^ against ALL reflections. The weighted *R*-factor *wR* and goodness of fit *S* are based on *F*^2^, conventional *R*-factors *R* are based on *F*, with *F* set to zero for negative *F*^2^. The threshold expression of *F*^2^ > σ(*F*^2^) is used only for calculating *R*-factors(gt) *etc*. and is not relevant to the choice of reflections for refinement. *R*-factors based on *F*^2^ are statistically about twice as large as those based on *F*, and *R*- factors based on ALL data will be even larger.

### Fractional atomic coordinates and isotropic or equivalent isotropic displacement parameters (Å^2^)

**Table:**

	*x*	*y*	*z*	*U*_iso_*/*U*_eq_	
C1	1.1950 (11)	0.7924 (9)	0.6895 (2)	0.0430 (16)	
H1A	1.2029	0.6618	0.6795	0.052*	
H1B	1.3132	0.8197	0.6710	0.052*	
C2	1.2449 (11)	0.7675 (9)	0.7428 (2)	0.0458 (16)	
H2A	1.1365	0.7263	0.7613	0.055*	
H2B	1.3990	0.6569	0.7483	0.055*	
C3	1.2281 (10)	0.9646 (9)	0.7610 (2)	0.0396 (14)	
H3A	1.3493	0.9959	0.7452	0.047*	
H3B	1.2542	0.9433	0.7960	0.047*	
C4	0.9981 (10)	1.1510 (8)	0.7517 (2)	0.0371 (14)	
C5	0.9477 (9)	1.1644 (8)	0.6975 (2)	0.0312 (12)	
H5	1.0720	1.1892	0.6803	0.037*	
C6	0.7297 (10)	1.3533 (8)	0.6823 (2)	0.0387 (14)	
H6A	0.5968	1.3321	0.6948	0.046*	
H6B	0.7236	1.4759	0.6966	0.046*	
C7	0.7201 (10)	1.3899 (8)	0.6279 (2)	0.0380 (14)	
H7A	0.8453	1.4242	0.6163	0.046*	
H7B	0.5764	1.5103	0.6196	0.046*	
C8	0.7368 (9)	1.2052 (7)	0.6010 (2)	0.0298 (12)	
C9	0.9473 (9)	1.0093 (8)	0.6205 (2)	0.0303 (12)	
H9	1.0819	1.0340	0.6101	0.036*	
C10	0.9619 (9)	0.9668 (8)	0.6769 (2)	0.0325 (13)	
C11	0.9744 (11)	0.8186 (8)	0.5939 (2)	0.0395 (14)	
H11A	1.1158	0.6994	0.6042	0.047*	
H11B	0.8468	0.7848	0.6036	0.047*	
C12	0.9811 (11)	0.8525 (8)	0.5399 (2)	0.0408 (15)	
H12A	0.9805	0.7332	0.5254	0.049*	
H12B	1.1236	0.8609	0.5297	0.049*	
C13	0.7792 (9)	1.0501 (8)	0.5211 (2)	0.0294 (12)	
H13	0.6422	1.0362	0.5348	0.035*	
C14	0.7750 (9)	1.2403 (7)	0.5449 (2)	0.0307 (13)	
C15	0.5757 (10)	1.4435 (8)	0.5244 (2)	0.0357 (14)	
H15A	0.4321	1.4525	0.5397	0.043*	
H15B	0.5972	1.5619	0.5338	0.043*	
C16	0.5533 (10)	1.4622 (8)	0.4699 (2)	0.0365 (14)	
H16A	0.4172	1.5882	0.4602	0.044*	
H16B	0.6874	1.4710	0.4538	0.044*	
C17	0.5338 (9)	1.2735 (8)	0.4552 (2)	0.0323 (13)	
H17	0.4139	1.2615	0.4772	0.039*	
C18	0.7506 (9)	1.0735 (8)	0.4665 (2)	0.0332 (13)	
C19	0.6834 (12)	0.9156 (9)	0.4482 (2)	0.0423 (15)	
H19A	0.5848	0.8828	0.4723	0.051*	
H19B	0.8191	0.7873	0.4417	0.051*	
C20	0.5545 (11)	1.0187 (9)	0.4013 (2)	0.0412 (14)	
H20A	0.4261	0.9839	0.3987	0.049*	
H20B	0.6567	0.9708	0.3728	0.049*	
C21	0.4672 (10)	1.2545 (8)	0.4038 (2)	0.0340 (13)	
H21	0.2983	1.3161	0.4037	0.041*	
C22	0.5414 (10)	1.3552 (9)	0.3616 (2)	0.0406 (14)	
C23	0.4498 (12)	1.5885 (9)	0.3589 (2)	0.0454 (16)	
H23A	0.3236	1.6452	0.3821	0.055*	
H23B	0.3969	1.6443	0.3264	0.055*	
H23C	0.5699	1.6257	0.3664	0.055*	
C24	1.0309 (13)	1.3405 (10)	0.7627 (3)	0.0529 (18)	
H24A	0.8830	1.4589	0.7652	0.064*	
H24B	1.1215	1.3702	0.7367	0.064*	
H24C	1.1091	1.3127	0.7932	0.064*	
C25	0.8197 (11)	1.1357 (10)	0.7870 (2)	0.0465 (16)	
H25A	0.6710	1.2459	0.7788	0.056*	
H25B	0.8558	1.1490	0.8197	0.056*	
H25C	0.8174	1.0023	0.7854	0.056*	
C26	0.5133 (10)	1.1867 (9)	0.6118 (2)	0.0393 (14)	
H26A	0.5423	1.0421	0.6124	0.047*	
H26B	0.4052	1.2659	0.5867	0.047*	
H26C	0.4504	1.2404	0.6431	0.047*	
C27	0.7810 (11)	0.9017 (10)	0.6983 (2)	0.0432 (15)	
H27A	0.6384	1.0240	0.7028	0.052*	
H27B	0.8304	0.8252	0.7294	0.052*	
H27C	0.7588	0.8141	0.6764	0.052*	
C28	0.9935 (10)	1.2664 (9)	0.5329 (2)	0.0390 (14)	
H28A	0.9859	1.3310	0.5003	0.047*	
H28B	1.1242	1.1315	0.5348	0.047*	
H28C	1.0090	1.3534	0.5560	0.047*	
C29	0.9562 (10)	1.0664 (9)	0.4360 (2)	0.0415 (15)	
H29A	0.9402	1.0422	0.4027	0.050*	
H29B	1.0945	0.9549	0.4491	0.050*	
H29C	0.9649	1.1975	0.4367	0.050*	
O30	0.6564 (8)	1.2631 (6)	0.32799 (17)	0.0533 (12)	
C31	0.2889 (11)	0.9966 (8)	−0.0337 (2)	0.0380 (14)	
H31A	0.1559	1.1231	−0.0249	0.046*	
H31B	0.4187	0.9845	−0.0154	0.046*	
C32	0.3420 (11)	1.0165 (8)	−0.0873 (2)	0.0397 (15)	
H32A	0.2091	1.0375	−0.1058	0.048*	
H32B	0.3727	1.1381	−0.0944	0.048*	
C33	0.5454 (10)	0.8262 (9)	−0.1030 (2)	0.0390 (14)	
H33A	0.6810	0.8159	−0.0872	0.047*	
H33B	0.5695	0.8421	−0.1382	0.047*	
C34	0.5221 (10)	0.6230 (8)	−0.0909 (2)	0.0359 (14)	
C35	0.4492 (10)	0.6168 (8)	−0.0372 (2)	0.0304 (12)	
H35	0.5796	0.6082	−0.0190	0.037*	
C36	0.4218 (10)	0.4208 (8)	−0.0203 (2)	0.0373 (14)	
H36A	0.2818	0.4289	−0.0335	0.045*	
H36B	0.5510	0.3000	−0.0329	0.045*	
C37	0.4107 (10)	0.3926 (8)	0.0342 (2)	0.0339 (13)	
H37A	0.5570	0.3714	0.0470	0.041*	
H37B	0.3895	0.2671	0.0433	0.041*	
C38	0.2185 (9)	0.5761 (8)	0.0580 (2)	0.0298 (12)	
C39	0.2278 (9)	0.7806 (7)	0.0369 (2)	0.0304 (12)	
H39	0.3739	0.7714	0.0479	0.037*	
C40	0.2399 (9)	0.8093 (7)	−0.0192 (2)	0.0294 (12)	
C41	0.0424 (10)	0.9667 (8)	0.0609 (2)	0.0361 (13)	
H41A	0.0621	1.0914	0.0498	0.043*	
H41B	−0.1072	0.9866	0.0504	0.043*	
C42	0.0455 (11)	0.9435 (8)	0.1160 (2)	0.0390 (14)	
H42A	0.1831	0.9488	0.1269	0.047*	
H42B	−0.0874	1.0603	0.1290	0.047*	
C43	0.0415 (9)	0.7436 (7)	0.1361 (2)	0.0297 (12)	
H43	−0.0941	0.7429	0.1220	0.036*	
C44	0.2496 (9)	0.5564 (8)	0.1153 (2)	0.0293 (12)	
C45	0.2555 (10)	0.3504 (8)	0.1369 (2)	0.0349 (13)	
H45A	0.1406	0.3254	0.1209	0.042*	
H45B	0.4053	0.2385	0.1292	0.042*	
C46	0.2121 (10)	0.3379 (8)	0.1917 (2)	0.0333 (13)	
H46A	0.2036	0.2075	0.2020	0.040*	
H46B	0.3367	0.3431	0.2088	0.040*	
C47	−0.0109 (9)	0.5200 (8)	0.2032 (2)	0.0305 (12)	
H47	−0.1218	0.5185	0.1805	0.037*	
C48	0.0036 (9)	0.7260 (8)	0.1906 (2)	0.0319 (13)	
C49	−0.2381 (10)	0.8751 (9)	0.2069 (2)	0.0394 (14)	
H49A	−0.2466	1.0120	0.2114	0.047*	
H49B	−0.3495	0.8906	0.1827	0.047*	
C50	−0.2841 (10)	0.7765 (9)	0.2548 (2)	0.0418 (15)	
H50A	−0.4453	0.8038	0.2580	0.050*	
H50B	−0.2466	0.8327	0.2823	0.050*	
C51	−0.1296 (10)	0.5406 (9)	0.2538 (2)	0.0365 (13)	
H51	−0.2332	0.4731	0.2533	0.044*	
C52	0.0131 (10)	0.4465 (9)	0.2969 (2)	0.0397 (14)	
C53	0.1511 (11)	0.2139 (9)	0.3016 (2)	0.0459 (16)	
H53A	0.0815	0.1494	0.2829	0.055*	
H53B	0.1549	0.1647	0.3355	0.055*	
H53C	0.3057	0.1781	0.2893	0.055*	
C54	0.7616 (11)	0.4468 (10)	−0.0980 (2)	0.0480 (17)	
H54A	0.7539	0.3146	−0.0938	0.058*	
H54B	0.8628	0.4512	−0.0743	0.058*	
H54C	0.8197	0.4632	−0.1305	0.058*	
C55	0.3667 (12)	0.6029 (10)	−0.1267 (2)	0.0476 (16)	
H55A	0.3383	0.4834	−0.1166	0.057*	
H55B	0.4395	0.5851	−0.1588	0.057*	
H55C	0.2230	0.7272	−0.1278	0.057*	
C56	−0.0049 (10)	0.5719 (9)	0.0467 (2)	0.0384 (14)	
H56A	−0.1281	0.7110	0.0474	0.046*	
H56B	−0.0364	0.4806	0.0708	0.046*	
H56C	0.0072	0.5216	0.0147	0.046*	
C57	0.0192 (10)	0.8489 (9)	−0.0428 (2)	0.0405 (14)	
H57A	0.0109	0.7182	−0.0448	0.049*	
H57B	0.0149	0.9139	−0.0752	0.049*	
H57C	−0.1094	0.9398	−0.0234	0.049*	
C58	0.4763 (10)	0.5523 (9)	0.1273 (2)	0.0372 (14)	
H58A	0.5085	0.5060	0.1612	0.045*	
H58B	0.4678	0.6905	0.1214	0.045*	
H58C	0.5971	0.4576	0.1070	0.045*	
C59	0.1696 (10)	0.7535 (9)	0.2215 (2)	0.0386 (14)	
H59A	0.1122	0.7685	0.2548	0.046*	
H59B	0.1872	0.8767	0.2093	0.046*	
H59C	0.3165	0.6331	0.2201	0.046*	
O60	0.0185 (8)	0.5464 (7)	0.32916 (17)	0.0546 (12)	

### Atomic displacement parameters (Å^2^)

**Table:**

	*U*^11^	*U*^22^	*U*^33^	*U*^12^	*U*^13^	*U*^23^
C1	0.048 (4)	0.027 (3)	0.034 (3)	0.001 (3)	0.000 (3)	0.000 (2)
C2	0.045 (4)	0.038 (4)	0.038 (4)	−0.001 (3)	−0.003 (3)	−0.006 (3)
C3	0.043 (4)	0.038 (3)	0.033 (3)	−0.014 (3)	−0.002 (3)	0.001 (3)
C4	0.043 (3)	0.025 (3)	0.043 (4)	−0.014 (3)	0.001 (3)	−0.001 (3)
C5	0.030 (3)	0.023 (3)	0.037 (3)	−0.008 (2)	0.003 (2)	−0.007 (2)
C6	0.046 (4)	0.018 (3)	0.040 (3)	−0.002 (3)	0.000 (3)	−0.003 (2)
C7	0.045 (4)	0.017 (3)	0.043 (3)	−0.004 (3)	−0.001 (3)	−0.005 (2)
C8	0.031 (3)	0.013 (2)	0.041 (3)	−0.005 (2)	0.000 (2)	−0.003 (2)
C9	0.030 (3)	0.021 (3)	0.037 (3)	−0.009 (2)	0.001 (2)	−0.001 (2)
C10	0.038 (3)	0.022 (3)	0.035 (3)	−0.011 (3)	0.003 (2)	−0.004 (2)
C11	0.046 (3)	0.016 (3)	0.046 (4)	−0.003 (2)	−0.001 (3)	−0.007 (2)
C12	0.045 (4)	0.019 (3)	0.043 (4)	0.000 (3)	−0.005 (3)	−0.005 (2)
C13	0.029 (3)	0.016 (3)	0.038 (3)	−0.005 (2)	−0.002 (2)	−0.001 (2)
C14	0.034 (3)	0.012 (2)	0.038 (3)	−0.003 (2)	−0.003 (2)	−0.003 (2)
C15	0.040 (3)	0.013 (3)	0.044 (3)	−0.003 (2)	−0.006 (3)	0.001 (2)
C16	0.040 (3)	0.017 (3)	0.044 (3)	−0.005 (2)	−0.004 (3)	−0.003 (2)
C17	0.032 (3)	0.024 (3)	0.041 (3)	−0.012 (2)	−0.004 (2)	0.000 (2)
C18	0.038 (3)	0.017 (3)	0.041 (3)	−0.010 (2)	−0.001 (3)	−0.002 (2)
C19	0.065 (4)	0.022 (3)	0.042 (4)	−0.021 (3)	−0.004 (3)	−0.001 (3)
C20	0.061 (4)	0.031 (3)	0.038 (3)	−0.026 (3)	0.000 (3)	−0.004 (3)
C21	0.035 (3)	0.022 (3)	0.043 (3)	−0.012 (2)	0.003 (3)	−0.004 (2)
C22	0.045 (4)	0.037 (3)	0.042 (4)	−0.019 (3)	−0.001 (3)	−0.004 (3)
C23	0.059 (4)	0.028 (3)	0.053 (4)	−0.022 (3)	−0.002 (3)	0.001 (3)
C24	0.077 (5)	0.041 (4)	0.049 (4)	−0.033 (4)	−0.009 (4)	−0.002 (3)
C25	0.053 (4)	0.037 (3)	0.042 (4)	−0.012 (3)	0.008 (3)	−0.011 (3)
C26	0.033 (3)	0.035 (3)	0.043 (4)	−0.009 (3)	0.000 (3)	−0.001 (3)
C27	0.052 (4)	0.034 (3)	0.044 (4)	−0.019 (3)	0.002 (3)	0.000 (3)
C28	0.039 (3)	0.036 (3)	0.043 (4)	−0.018 (3)	−0.003 (3)	0.002 (3)
C29	0.041 (3)	0.035 (3)	0.038 (3)	−0.008 (3)	0.007 (3)	−0.006 (3)
O30	0.069 (3)	0.037 (2)	0.049 (3)	−0.020 (2)	0.011 (2)	−0.002 (2)
C31	0.054 (4)	0.021 (3)	0.039 (3)	−0.016 (3)	−0.001 (3)	−0.002 (2)
C32	0.060 (4)	0.018 (3)	0.042 (4)	−0.018 (3)	−0.004 (3)	−0.001 (2)
C33	0.043 (4)	0.035 (3)	0.037 (3)	−0.015 (3)	−0.004 (3)	−0.002 (3)
C34	0.043 (3)	0.025 (3)	0.036 (3)	−0.011 (3)	−0.001 (3)	−0.004 (2)
C35	0.035 (3)	0.021 (3)	0.035 (3)	−0.011 (2)	−0.004 (2)	−0.002 (2)
C36	0.047 (4)	0.014 (3)	0.047 (4)	−0.010 (3)	0.003 (3)	−0.005 (2)
C37	0.041 (3)	0.016 (3)	0.038 (3)	−0.007 (2)	0.002 (3)	−0.001 (2)
C38	0.033 (3)	0.015 (3)	0.038 (3)	−0.007 (2)	−0.004 (2)	−0.001 (2)
C39	0.034 (3)	0.015 (3)	0.041 (3)	−0.008 (2)	−0.002 (2)	−0.005 (2)
C40	0.034 (3)	0.013 (3)	0.039 (3)	−0.008 (2)	−0.001 (2)	−0.004 (2)
C41	0.045 (3)	0.015 (3)	0.043 (3)	−0.009 (2)	0.008 (3)	−0.002 (2)
C42	0.052 (4)	0.017 (3)	0.045 (4)	−0.012 (3)	0.009 (3)	−0.008 (2)
C43	0.031 (3)	0.014 (3)	0.041 (3)	−0.009 (2)	−0.002 (2)	0.001 (2)
C44	0.030 (3)	0.013 (2)	0.041 (3)	−0.006 (2)	0.002 (2)	0.000 (2)
C45	0.041 (3)	0.019 (3)	0.041 (3)	−0.011 (2)	0.002 (3)	−0.006 (2)
C46	0.036 (3)	0.020 (3)	0.041 (3)	−0.010 (2)	−0.001 (2)	0.002 (2)
C47	0.033 (3)	0.021 (3)	0.034 (3)	−0.009 (2)	0.000 (2)	−0.001 (2)
C48	0.032 (3)	0.019 (3)	0.044 (3)	−0.010 (2)	0.000 (2)	−0.005 (2)
C49	0.039 (3)	0.027 (3)	0.044 (4)	−0.006 (3)	0.004 (3)	−0.006 (3)
C50	0.035 (3)	0.033 (3)	0.045 (4)	−0.004 (3)	0.004 (3)	−0.004 (3)
C51	0.034 (3)	0.029 (3)	0.046 (4)	−0.014 (3)	0.001 (3)	0.001 (3)
C52	0.043 (3)	0.038 (3)	0.034 (3)	−0.015 (3)	0.006 (3)	−0.004 (3)
C53	0.051 (4)	0.027 (3)	0.045 (4)	−0.006 (3)	−0.003 (3)	0.008 (3)
C54	0.052 (4)	0.032 (3)	0.045 (4)	−0.005 (3)	0.004 (3)	−0.003 (3)
C55	0.078 (5)	0.032 (3)	0.038 (3)	−0.028 (3)	−0.008 (3)	−0.002 (3)
C56	0.052 (4)	0.025 (3)	0.040 (3)	−0.019 (3)	0.000 (3)	0.001 (3)
C57	0.044 (4)	0.029 (3)	0.044 (4)	−0.011 (3)	−0.005 (3)	0.000 (3)
C58	0.037 (3)	0.036 (3)	0.035 (3)	−0.013 (3)	−0.001 (2)	−0.005 (3)
C59	0.041 (3)	0.029 (3)	0.049 (4)	−0.018 (3)	−0.001 (3)	−0.005 (3)
O60	0.063 (3)	0.042 (3)	0.052 (3)	−0.014 (2)	−0.017 (2)	−0.006 (2)

### Geometric parameters (Å, °)

**Table:**

C1—C2	1.522 (8)	C31—C32	1.523 (8)
C1—C10	1.539 (8)	C31—C40	1.546 (8)
C1—H1A	0.9900	C31—H31A	0.9900
C1—H1B	0.9900	C31—H31B	0.9900
C2—C3	1.516 (9)	C32—C33	1.518 (8)
C2—H2A	0.9900	C32—H32A	0.9900
C2—H2B	0.9900	C32—H32B	0.9900
C3—C4	1.543 (8)	C33—C34	1.555 (8)
C3—H3A	0.9900	C33—H33A	0.9900
C3—H3B	0.9900	C33—H33B	0.9900
C4—C25	1.522 (9)	C34—C55	1.534 (9)
C4—C24	1.542 (8)	C34—C35	1.547 (8)
C4—C5	1.553 (8)	C34—C54	1.549 (8)
C5—C6	1.539 (8)	C35—C36	1.542 (8)
C5—C10	1.556 (7)	C35—C40	1.566 (7)
C5—H5	1.0000	C35—H35	1.0000
C6—C7	1.519 (8)	C36—C37	1.516 (8)
C6—H6A	0.9900	C36—H36A	0.9900
C6—H6B	0.9900	C36—H36B	0.9900
C7—C8	1.553 (7)	C37—C38	1.546 (7)
C7—H7A	0.9900	C37—H37A	0.9900
C7—H7B	0.9900	C37—H37B	0.9900
C8—C26	1.543 (8)	C38—C56	1.536 (8)
C8—C9	1.571 (7)	C38—C39	1.574 (7)
C8—C14	1.588 (8)	C38—C44	1.610 (8)
C9—C11	1.564 (7)	C39—C41	1.542 (7)
C9—C10	1.577 (8)	C39—C40	1.563 (8)
C9—H9	1.0000	C39—H39	1.0000
C10—C27	1.534 (8)	C40—C57	1.530 (8)
C11—C12	1.507 (8)	C41—C42	1.533 (8)
C11—H11A	0.9900	C41—H41A	0.9900
C11—H11B	0.9900	C41—H41B	0.9900
C12—C13	1.544 (7)	C42—C43	1.520 (7)
C12—H12A	0.9900	C42—H42A	0.9900
C12—H12B	0.9900	C42—H42B	0.9900
C13—C18	1.531 (8)	C43—C48	1.529 (8)
C13—C14	1.573 (7)	C43—C44	1.572 (7)
C13—H13	1.0000	C43—H43	1.0000
C14—C28	1.544 (8)	C44—C58	1.536 (8)
C14—C15	1.572 (7)	C44—C45	1.548 (7)
C15—C16	1.526 (8)	C45—C46	1.540 (8)
C15—H15A	0.9900	C45—H45A	0.9900
C15—H15B	0.9900	C45—H45B	0.9900
C16—C17	1.523 (8)	C46—C47	1.524 (7)
C16—H16A	0.9900	C46—H46A	0.9900
C16—H16B	0.9900	C46—H46B	0.9900
C17—C18	1.550 (8)	C47—C48	1.548 (8)
C17—C21	1.559 (8)	C47—C51	1.558 (8)
C17—H17	1.0000	C47—H47	1.0000
C18—C19	1.532 (8)	C48—C59	1.521 (8)
C18—C29	1.538 (8)	C48—C49	1.548 (8)
C19—C20	1.551 (9)	C49—C50	1.538 (9)
C19—H19A	0.9900	C49—H49A	0.9900
C19—H19B	0.9900	C49—H49B	0.9900
C20—C21	1.556 (8)	C50—C51	1.564 (8)
C20—H20A	0.9900	C50—H50A	0.9900
C20—H20B	0.9900	C50—H50B	0.9900
C21—C22	1.501 (8)	C51—C52	1.492 (9)
C21—H21	1.0000	C51—H51	1.0000
C22—O30	1.216 (7)	C52—O60	1.217 (7)
C22—C23	1.525 (9)	C52—C53	1.525 (8)
C23—H23A	0.9800	C53—H53A	0.9800
C23—H23B	0.9800	C53—H53B	0.9800
C23—H23C	0.9800	C53—H53C	0.9800
C24—H24A	0.9800	C54—H54A	0.9800
C24—H24B	0.9800	C54—H54B	0.9800
C24—H24C	0.9800	C54—H54C	0.9800
C25—H25A	0.9800	C55—H55A	0.9800
C25—H25B	0.9800	C55—H55B	0.9800
C25—H25C	0.9800	C55—H55C	0.9800
C26—H26A	0.9800	C56—H56A	0.9800
C26—H26B	0.9800	C56—H56B	0.9800
C26—H26C	0.9800	C56—H56C	0.9800
C27—H27A	0.9800	C57—H57A	0.9800
C27—H27B	0.9800	C57—H57B	0.9800
C27—H27C	0.9800	C57—H57C	0.9800
C28—H28A	0.9800	C58—H58A	0.9800
C28—H28B	0.9800	C58—H58B	0.9800
C28—H28C	0.9800	C58—H58C	0.9800
C29—H29A	0.9800	C59—H59A	0.9800
C29—H29B	0.9800	C59—H59B	0.9800
C29—H29C	0.9800	C59—H59C	0.9800
			
C2—C1—C10	113.4 (5)	C32—C31—C40	113.0 (5)
C2—C1—H1A	108.9	C32—C31—H31A	109.0
C10—C1—H1A	108.9	C40—C31—H31A	109.0
C2—C1—H1B	108.9	C32—C31—H31B	109.0
C10—C1—H1B	108.9	C40—C31—H31B	109.0
H1A—C1—H1B	107.7	H31A—C31—H31B	107.8
C3—C2—C1	111.7 (5)	C33—C32—C31	111.3 (5)
C3—C2—H2A	109.3	C33—C32—H32A	109.4
C1—C2—H2A	109.3	C31—C32—H32A	109.4
C3—C2—H2B	109.3	C33—C32—H32B	109.4
C1—C2—H2B	109.3	C31—C32—H32B	109.4
H2A—C2—H2B	107.9	H32A—C32—H32B	108.0
C2—C3—C4	113.7 (5)	C32—C33—C34	114.0 (5)
C2—C3—H3A	108.8	C32—C33—H33A	108.8
C4—C3—H3A	108.8	C34—C33—H33A	108.8
C2—C3—H3B	108.8	C32—C33—H33B	108.8
C4—C3—H3B	108.8	C34—C33—H33B	108.8
H3A—C3—H3B	107.7	H33A—C33—H33B	107.6
C25—C4—C24	107.7 (5)	C55—C34—C35	115.7 (5)
C25—C4—C3	109.8 (5)	C55—C34—C54	107.7 (5)
C24—C4—C3	105.7 (5)	C35—C34—C54	108.9 (5)
C25—C4—C5	116.0 (5)	C55—C34—C33	110.1 (5)
C24—C4—C5	109.5 (5)	C35—C34—C33	108.0 (4)
C3—C4—C5	107.7 (4)	C54—C34—C33	106.1 (5)
C6—C5—C4	113.3 (4)	C36—C35—C34	113.3 (4)
C6—C5—C10	111.2 (5)	C36—C35—C40	109.5 (4)
C4—C5—C10	117.1 (4)	C34—C35—C40	117.6 (4)
C6—C5—H5	104.6	C36—C35—H35	105.1
C4—C5—H5	104.6	C34—C35—H35	105.1
C10—C5—H5	104.6	C40—C35—H35	105.1
C7—C6—C5	111.0 (4)	C37—C36—C35	111.8 (5)
C7—C6—H6A	109.4	C37—C36—H36A	109.3
C5—C6—H6A	109.4	C35—C36—H36A	109.3
C7—C6—H6B	109.4	C37—C36—H36B	109.3
C5—C6—H6B	109.4	C35—C36—H36B	109.3
H6A—C6—H6B	108.0	H36A—C36—H36B	107.9
C6—C7—C8	114.5 (5)	C36—C37—C38	113.7 (5)
C6—C7—H7A	108.6	C36—C37—H37A	108.8
C8—C7—H7A	108.6	C38—C37—H37A	108.8
C6—C7—H7B	108.6	C36—C37—H37B	108.8
C8—C7—H7B	108.6	C38—C37—H37B	108.8
H7A—C7—H7B	107.6	H37A—C37—H37B	107.7
C26—C8—C7	106.9 (5)	C56—C38—C37	107.0 (4)
C26—C8—C9	112.2 (4)	C56—C38—C39	111.7 (4)
C7—C8—C9	107.6 (4)	C37—C38—C39	108.9 (4)
C26—C8—C14	111.1 (4)	C56—C38—C44	110.4 (4)
C7—C8—C14	110.7 (4)	C37—C38—C44	110.5 (4)
C9—C8—C14	108.3 (4)	C39—C38—C44	108.4 (4)
C11—C9—C8	109.8 (4)	C41—C39—C40	114.1 (4)
C11—C9—C10	113.0 (4)	C41—C39—C38	110.3 (4)
C8—C9—C10	116.5 (4)	C40—C39—C38	115.8 (4)
C11—C9—H9	105.5	C41—C39—H39	105.2
C8—C9—H9	105.5	C40—C39—H39	105.2
C10—C9—H9	105.5	C38—C39—H39	105.2
C27—C10—C1	107.8 (5)	C57—C40—C31	107.3 (4)
C27—C10—C5	113.3 (5)	C57—C40—C39	113.0 (5)
C1—C10—C5	107.2 (5)	C31—C40—C39	109.1 (4)
C27—C10—C9	112.4 (5)	C57—C40—C35	113.4 (4)
C1—C10—C9	109.2 (4)	C31—C40—C35	106.4 (4)
C5—C10—C9	106.7 (4)	C39—C40—C35	107.3 (4)
C12—C11—C9	113.2 (5)	C42—C41—C39	113.4 (5)
C12—C11—H11A	108.9	C42—C41—H41A	108.9
C9—C11—H11A	108.9	C39—C41—H41A	108.9
C12—C11—H11B	108.9	C42—C41—H41B	108.9
C9—C11—H11B	108.9	C39—C41—H41B	108.9
H11A—C11—H11B	107.8	H41A—C41—H41B	107.7
C11—C12—C13	112.6 (5)	C43—C42—C41	112.5 (4)
C11—C12—H12A	109.1	C43—C42—H42A	109.1
C13—C12—H12A	109.1	C41—C42—H42A	109.1
C11—C12—H12B	109.1	C43—C42—H42B	109.1
C13—C12—H12B	109.1	C41—C42—H42B	109.1
H12A—C12—H12B	107.8	H42A—C42—H42B	107.8
C18—C13—C12	115.3 (4)	C42—C43—C48	115.4 (4)
C18—C13—C14	116.3 (4)	C42—C43—C44	109.8 (4)
C12—C13—C14	109.6 (4)	C48—C43—C44	115.1 (4)
C18—C13—H13	104.8	C42—C43—H43	105.1
C12—C13—H13	104.8	C48—C43—H43	105.1
C14—C13—H13	104.8	C44—C43—H43	105.1
C28—C14—C15	106.0 (5)	C58—C44—C45	106.2 (5)
C28—C14—C13	111.7 (4)	C58—C44—C43	112.3 (4)
C15—C14—C13	110.0 (4)	C45—C44—C43	110.9 (4)
C28—C14—C8	112.5 (4)	C58—C44—C38	111.2 (4)
C15—C14—C8	110.2 (4)	C45—C44—C38	109.8 (4)
C13—C14—C8	106.5 (4)	C43—C44—C38	106.5 (4)
C16—C15—C14	115.1 (4)	C46—C45—C44	115.4 (4)
C16—C15—H15A	108.5	C46—C45—H45A	108.4
C14—C15—H15A	108.5	C44—C45—H45A	108.4
C16—C15—H15B	108.5	C46—C45—H45B	108.4
C14—C15—H15B	108.5	C44—C45—H45B	108.4
H15A—C15—H15B	107.5	H45A—C45—H45B	107.5
C17—C16—C15	108.3 (5)	C47—C46—C45	107.7 (4)
C17—C16—H16A	110.0	C47—C46—H46A	110.2
C15—C16—H16A	110.0	C45—C46—H46A	110.2
C17—C16—H16B	110.0	C47—C46—H46B	110.2
C15—C16—H16B	110.0	C45—C46—H46B	110.2
H16A—C16—H16B	108.4	H46A—C46—H46B	108.5
C16—C17—C18	111.8 (5)	C46—C47—C48	111.2 (5)
C16—C17—C21	123.3 (5)	C46—C47—C51	122.5 (5)
C18—C17—C21	105.4 (4)	C48—C47—C51	105.6 (4)
C16—C17—H17	104.9	C46—C47—H47	105.4
C18—C17—H17	104.9	C48—C47—H47	105.4
C21—C17—H17	104.9	C51—C47—H47	105.4
C13—C18—C19	113.1 (5)	C59—C48—C43	116.4 (5)
C13—C18—C29	115.6 (5)	C59—C48—C49	107.7 (5)
C19—C18—C29	107.4 (5)	C43—C48—C49	112.4 (5)
C13—C18—C17	107.4 (4)	C59—C48—C47	112.8 (5)
C19—C18—C17	100.1 (5)	C43—C48—C47	107.2 (4)
C29—C18—C17	112.2 (5)	C49—C48—C47	99.0 (4)
C18—C19—C20	105.3 (4)	C50—C49—C48	105.3 (5)
C18—C19—H19A	110.7	C50—C49—H49A	110.7
C20—C19—H19A	110.7	C48—C49—H49A	110.7
C18—C19—H19B	110.7	C50—C49—H49B	110.7
C20—C19—H19B	110.7	C48—C49—H49B	110.7
H19A—C19—H19B	108.8	H49A—C49—H49B	108.8
C19—C20—C21	106.9 (4)	C49—C50—C51	106.3 (5)
C19—C20—H20A	110.4	C49—C50—H50A	110.5
C21—C20—H20A	110.4	C51—C50—H50A	110.5
C19—C20—H20B	110.4	C49—C50—H50B	110.5
C21—C20—H20B	110.4	C51—C50—H50B	110.5
H20A—C20—H20B	108.6	H50A—C50—H50B	108.7
C22—C21—C20	113.7 (5)	C52—C51—C47	118.6 (5)
C22—C21—C17	118.5 (5)	C52—C51—C50	114.7 (5)
C20—C21—C17	103.1 (4)	C47—C51—C50	103.3 (4)
C22—C21—H21	107.0	C52—C51—H51	106.5
C20—C21—H21	107.0	C47—C51—H51	106.5
C17—C21—H21	107.0	C50—C51—H51	106.5
O30—C22—C21	123.7 (6)	O60—C52—C51	122.9 (6)
O30—C22—C23	118.4 (6)	O60—C52—C53	118.5 (6)
C21—C22—C23	117.7 (5)	C51—C52—C53	118.5 (5)
C22—C23—H23A	109.5	C52—C53—H53A	109.5
C22—C23—H23B	109.5	C52—C53—H53B	109.5
H23A—C23—H23B	109.5	H53A—C53—H53B	109.5
C22—C23—H23C	109.5	C52—C53—H53C	109.5
H23A—C23—H23C	109.5	H53A—C53—H53C	109.5
H23B—C23—H23C	109.5	H53B—C53—H53C	109.5
C4—C24—H24A	109.5	C34—C54—H54A	109.5
C4—C24—H24B	109.5	C34—C54—H54B	109.5
H24A—C24—H24B	109.5	H54A—C54—H54B	109.5
C4—C24—H24C	109.5	C34—C54—H54C	109.5
H24A—C24—H24C	109.5	H54A—C54—H54C	109.5
H24B—C24—H24C	109.5	H54B—C54—H54C	109.5
C4—C25—H25A	109.5	C34—C55—H55A	109.5
C4—C25—H25B	109.5	C34—C55—H55B	109.5
H25A—C25—H25B	109.5	H55A—C55—H55B	109.5
C4—C25—H25C	109.5	C34—C55—H55C	109.5
H25A—C25—H25C	109.5	H55A—C55—H55C	109.5
H25B—C25—H25C	109.5	H55B—C55—H55C	109.5
C8—C26—H26A	109.5	C38—C56—H56A	109.5
C8—C26—H26B	109.5	C38—C56—H56B	109.5
H26A—C26—H26B	109.5	H56A—C56—H56B	109.5
C8—C26—H26C	109.5	C38—C56—H56C	109.5
H26A—C26—H26C	109.5	H56A—C56—H56C	109.5
H26B—C26—H26C	109.5	H56B—C56—H56C	109.5
C10—C27—H27A	109.5	C40—C57—H57A	109.5
C10—C27—H27B	109.5	C40—C57—H57B	109.5
H27A—C27—H27B	109.5	H57A—C57—H57B	109.5
C10—C27—H27C	109.5	C40—C57—H57C	109.5
H27A—C27—H27C	109.5	H57A—C57—H57C	109.5
H27B—C27—H27C	109.5	H57B—C57—H57C	109.5
C14—C28—H28A	109.5	C44—C58—H58A	109.5
C14—C28—H28B	109.5	C44—C58—H58B	109.5
H28A—C28—H28B	109.5	H58A—C58—H58B	109.5
C14—C28—H28C	109.5	C44—C58—H58C	109.5
H28A—C28—H28C	109.5	H58A—C58—H58C	109.5
H28B—C28—H28C	109.5	H58B—C58—H58C	109.5
C18—C29—H29A	109.5	C48—C59—H59A	109.5
C18—C29—H29B	109.5	C48—C59—H59B	109.5
H29A—C29—H29B	109.5	H59A—C59—H59B	109.5
C18—C29—H29C	109.5	C48—C59—H59C	109.5
H29A—C29—H29C	109.5	H59A—C59—H59C	109.5
H29B—C29—H29C	109.5	H59B—C59—H59C	109.5
			
C10—C1—C2—C3	−56.8 (8)	C40—C31—C32—C33	−58.7 (7)
C1—C2—C3—C4	55.7 (7)	C31—C32—C33—C34	55.6 (7)
C2—C3—C4—C25	75.5 (7)	C32—C33—C34—C55	77.0 (6)
C2—C3—C4—C24	−168.6 (5)	C32—C33—C34—C35	−50.1 (6)
C2—C3—C4—C5	−51.6 (6)	C32—C33—C34—C54	−166.7 (5)
C25—C4—C5—C6	60.6 (7)	C55—C34—C35—C36	56.7 (6)
C24—C4—C5—C6	−61.6 (7)	C54—C34—C35—C36	−64.7 (6)
C3—C4—C5—C6	−176.0 (5)	C33—C34—C35—C36	−179.5 (5)
C25—C4—C5—C10	−71.0 (6)	C55—C34—C35—C40	−72.7 (6)
C24—C4—C5—C10	166.9 (5)	C54—C34—C35—C40	165.9 (5)
C3—C4—C5—C10	52.4 (6)	C33—C34—C35—C40	51.1 (6)
C4—C5—C6—C7	165.2 (5)	C34—C35—C36—C37	165.2 (5)
C10—C5—C6—C7	−60.4 (6)	C40—C35—C36—C37	−61.4 (6)
C5—C6—C7—C8	57.1 (7)	C35—C36—C37—C38	57.2 (7)
C6—C7—C8—C26	70.3 (6)	C36—C37—C38—C56	71.7 (6)
C6—C7—C8—C9	−50.4 (6)	C36—C37—C38—C39	−49.2 (6)
C6—C7—C8—C14	−168.6 (5)	C36—C37—C38—C44	−168.1 (5)
C26—C8—C9—C11	63.6 (6)	C56—C38—C39—C41	63.7 (6)
C7—C8—C9—C11	−179.1 (5)	C37—C38—C39—C41	−178.4 (5)
C14—C8—C9—C11	−59.3 (6)	C44—C38—C39—C41	−58.1 (6)
C26—C8—C9—C10	−66.4 (6)	C56—C38—C39—C40	−67.8 (6)
C7—C8—C9—C10	50.9 (6)	C37—C38—C39—C40	50.2 (6)
C14—C8—C9—C10	170.6 (4)	C44—C38—C39—C40	170.5 (4)
C2—C1—C10—C27	−69.1 (7)	C32—C31—C40—C57	−66.6 (6)
C2—C1—C10—C5	53.2 (7)	C32—C31—C40—C39	170.6 (5)
C2—C1—C10—C9	168.5 (5)	C32—C31—C40—C35	55.1 (6)
C6—C5—C10—C27	−66.9 (6)	C41—C39—C40—C57	−59.4 (6)
C4—C5—C10—C27	65.6 (7)	C38—C39—C40—C57	70.3 (6)
C6—C5—C10—C1	174.2 (5)	C41—C39—C40—C31	59.9 (6)
C4—C5—C10—C1	−53.2 (6)	C38—C39—C40—C31	−170.4 (4)
C6—C5—C10—C9	57.3 (6)	C41—C39—C40—C35	174.8 (4)
C4—C5—C10—C9	−170.1 (4)	C38—C39—C40—C35	−55.5 (6)
C11—C9—C10—C27	−58.8 (6)	C36—C35—C40—C57	−67.0 (6)
C8—C9—C10—C27	69.7 (6)	C34—C35—C40—C57	64.1 (6)
C11—C9—C10—C1	60.8 (6)	C36—C35—C40—C31	175.2 (4)
C8—C9—C10—C1	−170.7 (5)	C34—C35—C40—C31	−53.6 (6)
C11—C9—C10—C5	176.4 (5)	C36—C35—C40—C39	58.5 (5)
C8—C9—C10—C5	−55.1 (6)	C34—C35—C40—C39	−170.4 (5)
C8—C9—C11—C12	53.2 (7)	C40—C39—C41—C42	−175.2 (5)
C10—C9—C11—C12	−175.0 (5)	C38—C39—C41—C42	52.4 (6)
C9—C11—C12—C13	−52.3 (7)	C39—C41—C42—C43	−52.5 (7)
C11—C12—C13—C18	−169.1 (5)	C41—C42—C43—C48	−170.0 (5)
C11—C12—C13—C14	57.5 (7)	C41—C42—C43—C44	58.0 (6)
C18—C13—C14—C28	−72.6 (6)	C42—C43—C44—C58	58.9 (6)
C12—C13—C14—C28	60.4 (6)	C48—C43—C44—C58	−73.2 (6)
C18—C13—C14—C15	44.8 (6)	C42—C43—C44—C45	177.5 (5)
C12—C13—C14—C15	177.7 (5)	C48—C43—C44—C45	45.4 (6)
C18—C13—C14—C8	164.2 (4)	C42—C43—C44—C38	−63.0 (5)
C12—C13—C14—C8	−62.9 (6)	C48—C43—C44—C38	164.8 (4)
C26—C8—C14—C28	178.2 (5)	C56—C38—C44—C58	177.9 (5)
C7—C8—C14—C28	59.6 (6)	C37—C38—C44—C58	59.8 (6)
C9—C8—C14—C28	−58.2 (5)	C39—C38—C44—C58	−59.5 (5)
C26—C8—C14—C15	60.1 (5)	C56—C38—C44—C45	60.7 (5)
C7—C8—C14—C15	−58.5 (6)	C37—C38—C44—C45	−57.4 (6)
C9—C8—C14—C15	−176.2 (5)	C39—C38—C44—C45	−176.7 (4)
C26—C8—C14—C13	−59.2 (5)	C56—C38—C44—C43	−59.4 (5)
C7—C8—C14—C13	−177.7 (4)	C37—C38—C44—C43	−177.6 (4)
C9—C8—C14—C13	64.5 (5)	C39—C38—C44—C43	63.2 (5)
C28—C14—C15—C16	75.6 (6)	C58—C44—C45—C46	77.5 (6)
C13—C14—C15—C16	−45.3 (7)	C43—C44—C45—C46	−44.8 (6)
C8—C14—C15—C16	−162.4 (5)	C38—C44—C45—C46	−162.2 (4)
C14—C15—C16—C17	55.1 (7)	C44—C45—C46—C47	54.2 (6)
C15—C16—C17—C18	−63.6 (6)	C45—C46—C47—C48	−63.8 (6)
C15—C16—C17—C21	169.2 (5)	C45—C46—C47—C51	170.2 (5)
C12—C13—C18—C19	67.6 (7)	C42—C43—C48—C59	−56.4 (6)
C14—C13—C18—C19	−162.1 (5)	C44—C43—C48—C59	73.1 (6)
C12—C13—C18—C29	−56.8 (7)	C42—C43—C48—C49	68.5 (6)
C14—C13—C18—C29	73.4 (6)	C44—C43—C48—C49	−162.0 (5)
C12—C13—C18—C17	177.0 (5)	C42—C43—C48—C47	176.2 (5)
C14—C13—C18—C17	−52.7 (6)	C44—C43—C48—C47	−54.3 (6)
C16—C17—C18—C13	62.0 (6)	C46—C47—C48—C59	−65.3 (6)
C21—C17—C18—C13	−161.7 (4)	C51—C47—C48—C59	69.6 (6)
C16—C17—C18—C19	−179.8 (5)	C46—C47—C48—C43	64.2 (6)
C21—C17—C18—C19	−43.5 (5)	C51—C47—C48—C43	−160.9 (4)
C16—C17—C18—C29	−66.1 (6)	C46—C47—C48—C49	−178.9 (5)
C21—C17—C18—C29	70.2 (6)	C51—C47—C48—C49	−43.9 (5)
C13—C18—C19—C20	153.6 (5)	C59—C48—C49—C50	−75.5 (6)
C29—C18—C19—C20	−77.7 (6)	C43—C48—C49—C50	155.0 (5)
C17—C18—C19—C20	39.6 (6)	C47—C48—C49—C50	42.1 (6)
C18—C19—C20—C21	−22.0 (7)	C48—C49—C50—C51	−25.3 (6)
C19—C20—C21—C22	124.5 (5)	C46—C47—C51—C52	29.5 (8)
C19—C20—C21—C17	−5.1 (6)	C48—C47—C51—C52	−99.0 (6)
C16—C17—C21—C22	33.5 (8)	C46—C47—C51—C50	157.7 (5)
C18—C17—C21—C22	−96.4 (6)	C48—C47—C51—C50	29.2 (6)
C16—C17—C21—C20	160.1 (5)	C49—C50—C51—C52	128.2 (5)
C18—C17—C21—C20	30.2 (6)	C49—C50—C51—C47	−2.3 (6)
C20—C21—C22—O30	1.0 (9)	C47—C51—C52—O60	119.0 (7)
C17—C21—C22—O30	122.3 (6)	C50—C51—C52—O60	−3.6 (8)
C20—C21—C22—C23	175.4 (5)	C47—C51—C52—C53	−63.6 (7)
C17—C21—C22—C23	−63.3 (7)	C50—C51—C52—C53	173.9 (5)

### Hydrogen-bond geometry (Å, °)

**Table:**

*D*—H···*A*	*D*—H	H···*A*	*D*···*A*	*D*—H···*A*
C20—H20B···O30	0.99	2.37	2.832 (7)	108
C50—H50B···O60	0.99	2.39	2.837 (7)	107
